# *Pholiota nameko* Polysaccharides Protect against Ultraviolet A-Induced Photoaging by Regulating Matrix Metalloproteinases in Human Dermal Fibroblasts

**DOI:** 10.3390/antiox11040739

**Published:** 2022-04-08

**Authors:** His Lin, Kuan-Chen Cheng, Jer-An Lin, Liang-Po Hsieh, Chun-Hsu Chou, Yu-Ying Wang, Ping-Shan Lai, Po-Cheng Chu, Chang-Wei Hsieh

**Affiliations:** 1 Department of Food Science and Biotechnology, National Chung Hsing University, 145 Xingda Road, South Dist., Taichung City 40227, Taiwan; scarrcon@dragon.nchu.edu.tw (H.L.); 7109043409@mail.nchu.edu.tw (Y.-Y.W.); 2 Institute of Biotechnology, National Taiwan University, Taipei 10617, Taiwan; kccheng@ntu.edu.tw; 3 Graduate Institute of Food Science Technology, National Taiwan University, Taipei 10617, Taiwan; 4 Department of Optometry, Asia University, 500, Lioufeng Road, Wufeng, Taichung 41354, Taiwan; 5 Department of Medical Research, China Medical University Hospital, Taichung City 40402, Taiwan; 6 Graduate Institute of Food Safety, National Chung Hsing University, 145 Xingda Road, South Dist., Taichung City 40227, Taiwan; lja@nchu.edu.tw; 7 Department of Neurology, Cheng Ching General Hospital, Taichung 40764, Taiwan; 3635@ccgh.com.tw; 8 Dr Jou Biotech Co., Ltd., No. 21, Lugong S. 2nd Road, Lukang Township, Changhua County 50544, Taiwan; drjou.akira@drjou.com.tw; 9 Department of Chemistry, National Chung Hsing University, No. 145, Xingda Road, Taichung 40227, Taiwan; pslai@email.nchu.edu.tw (P.-S.L.); g106051177@mail.nchu.edu.tw (P.-C.C.)

**Keywords:** *Pholiota nameko* polysaccharides, ultraviolet-A, human dermal fibroblasts, oxidative damage, aging

## Abstract

Ultraviolet-A (UVA) exposure is a major cause of skin aging and can induce oxidative damage and accelerate skin wrinkling. Many natural polysaccharides exhibit a UV protective effect. In research on *Pholiota nameko* polysaccharides (PNPs), a natural macromolecular polysaccharide (4.4–333.487 kDa), studies have shown that PNPs can significantly decrease elastase activity to protect against UVA-induced aging in Hs68 human dermal fibroblasts. Cellular experiments in the present study indicated that PNPs can protect against UVA-induced oxidative damage in Hs68 cells by inhibiting the production of reactive oxygen species. Furthermore, PNPs significantly attenuated UVA-induced cell aging by decreasing the protein expression of matrix metalloproteinase 1, 3, and 9. Pretreatment of Hs68 cells with PNP-40, PNP-60, and PNP-80 before UVA irradiation increased protein expression of tissue inhibitor metalloproteinase 1 by 41%, 42%, and 56% relative to untreated cells. In conclusion, this study demonstrates that PNPs are a natural resource with potentially beneficial effects in protecting against UVA-induced skin aging.

## 1. Introduction

Mushrooms are widely used in medicine, cosmetics, and other health products [1]. Research on the chemical composition, nutritional value, and therapeutic properties of mushrooms has advanced considerably in recent years. Mushrooms have been shown to possess many biologically active molecules, such as β-glucans, triterpenoids, and antioxidants [2]. Polysaccharides are the most well-known derivatives of mushrooms, and numerous studies have reported that they exhibit various physiological effects, including immunomodulation, antitumor, anti-inflammatory, antiviral, and antihyperglycemic activity [3,4]. Recent studies on the antioxidant potential of mushroom polysaccharides have demonstrated their applicability in skincare. One study showed that polysaccharides extracted from *Xylaria nigripes* were noncytotoxic to HaCaT cells and significantly reduced the production of reactive oxygen species (ROS) in the cells [5]. Another study reported that enzymatically hydrolyzed *Trametes* versicolor polysaccharides increased the survival rate of human HaCaT cells and reduced the generation of ROS under AAPH-induced oxidative stress [6]. Aside from protection against oxidative damage, other studies have found that *Ganoderma lucidum* polysaccharides (GL-PS) can protect human dermal fibroblasts cells from upregulated concentrations of ROS and the expression of matrix metalloproteinase-1 expression (MMP-1), both of which are induced by exposure to ultraviolet-B (UVB). Polysaccharides of *Tremella fuciformis* have also been shown to promote collagen synthesis and protect mice against ultraviolet (UV) light and photoaging [7].

UV irradiation is the main external factor that causes skin aging and wrinkle formation [8,9]. Previous research showed that irradiating cells with UV light destroyed their morphology and induced apoptosis, which influenced extracellular matrix (ECM) production and secretion, leading to cellular malfunction [10]. Ultraviolet-A (UVA, 320–400 nm) accounts for up to 95% of UV radiation in the atmosphere and produces excessive ROS by stimulating NADPH oxidase enzyme activity [11,12]. UVA can penetrate the deeper layers of the dermis, and this has a direct impact on the cells and ECM of the dermis [9]. UVA irradiation activates different compartments of signaling pathways in the dermis, such as mitogen-activated protein kinases (MAPKs) and transcription protein nuclear factor (NF)-κB, which activate B cells. Studies have shown that MAPKs and NF-κB can further reinforce the expression of MMPs, thus accelerating collagen and elastin degradation [13,14].

*Pholiota nameko* is an edible, medicinal fungus predominantly cultured in China and Japan. Many studies have indicated that polysaccharides extracted from the fruiting body of *P. nameko* can exhibit immunoregulation, anti-inflammatory, antioxidant, antitumor, and antihyperlipidemic activity [15,16,17,18]. 

In a previous study, *P. nameko* polysaccharides (PNPs) extracted by ethanol precipitation at concentrations of 40%, 60%, 80% (*v*/*v*), referred to respectively as PNP-40, PNP-60, and PNP-80, demonstrated considerable antioxidant activity. Through chemical analysis, previous research found that the polysaccharide content of PNP-40, PNP-60, and PNP-80 was 45.1%, 78.0%, and 72.2%; β-glucan content was 20.2%, 12.2%, and 10.2%; protein content was 11.2%, 14.5%, and 26.9%; and uronic acid content was 5.9%, 7.7%, and 3.8%, respectively [19]. The PNPs in that study not only exhibited a predominantly protective effect against hydrogen peroxide (H_2_O_2_)-induced oxidative damage to L929 cells, but also promoted L929 cellular migration and proliferation, which might have potential in promoting wound healing. Moreover, the PNPs showed significant collagenase inhibitory activity, especially PNP-80, which had a more favorable inhibitory rate than epigallocatechin gallate (EGCG) [19], a polyphenol extracted from green tea (*Camellia sinensis*) that has also been found to be an inhibitor of collagenase and elastase [20]. However, few studies have investigated whether PNPs can protect human dermal fibroblasts against cellular photoaging resulting from UV irradiation [17,19]. Thus, the present study assessed their ability to inhibit elastase and collagenase activity in vitro, protect human dermal fibroblast Hs68 cells from UVA-induced cell damage, and reduce UVA-induced ROS overproduction. Furthermore, we also observed protein expression of MMP1, 3, 9 in Hs68 cells to explore whether PNPs can protect human fibroblasts against UVA-induced photoaging.

## 2. Materials and Methods

### 2.1. Chemicals

Fluorescein-labeled dye 2′,7′-dichlorofluorescein diacetate (DCF-DA); 3-(4,5-dimethylthiazol-2-yl)-2,5-diphenyltetrazolium bromide (MTT); elastase; pancreatic type I from porcine pancreas; and N-Succinyl-Ala-Ala-Ala-p-nitroanilide elastase substrate were purchased from Sigma-Aldrich (St. Louis, MO, USA). Retinoic acid (RA) was purchased from Alfa Aesar, Canada. Rabbit polyclonal antibodies against GAPDH; MMP-1, -3, -9; and anti-rabbit IgG secondary antibodies were purchased from Elabscience Biotechnology (HOU, USA). All other chemicals used in the experiments were of analytical grade.

### 2.2. Sample Preparation

*P. nameko* was purchased from the Rich Year Farm (Puli Township, Nantou County, Taiwan). PNPs were prepared as described in a previous study [17]. Briefly, PNPs were extracted using hot water and precipitated via graded ethanol precipitation. Ethanol was added at final concentrations of 40%, 60%, and 80% (*v*/*v*), and the PNPs precipitates were named PNP-40, PNP-60, and PNP-80, respectively. All PNPs samples were collected, lyophilized, and refrigerated at 4 °C.

### 2.3. Measurement of Elastase Activity

Elastase activity was measured following a previously described method but with some modification [21]. Briefly, 12.5 μL of elastase solution (1 μg/mL), 50 μL of 0.2 M Tris-HCl (pH = 8), and 25 μL of PNPs (125, 250, and 500 μg/mL) were mixed together in a 96-well plate and incubated at 25 °C for 15 min. Thereafter, 12.5 μL of 1.0 mM N-Succinyl-Ala-Ala-Ala-p-nitroanilide elastase substrate was added at 25 °C for 30 min. Finally, the absorbance of the samples was read at 410 nm using an ELISA reader. Distilled water was used as a control, and 25 μL of EGCG (125, 250, and 500 μg/mL) was used as a positive control.

Inhibitory rate of elastase (%) = [(Ac − Ats/Ac)] × 100%(1)

where Ac represents the absorbance of the control, and Ats represents the absorbance of the test sample.

### 2.4. Cell Culture and UVA Irradiation

Human dermal fibroblast cell line Hs68 (obtained from ATCC CRL-1635, Manassas, VA, USA) was cultured in Dulbecco’s modified Eagle’s medium (DMEM, Gibco^®^, Grand Island, NY, USA) containing 10% fetal bovine serum (FBS, Gibco^®^, Grand Island, NY, USA) and antibiotics (100 U penicillin and 100 U/mL streptomycin; Gibco^®^, Grand Island, NY, USA) under 5% CO_2_ at 37 °C. Cells were harvested after reaching confluence by using 0.05% trypsin–EDTA (Gibco^®^, Grand Island, NY, USA). Fresh culture medium was added to produce single-cell suspensions for further incubation. 

UVA irradiation was performed following a previously described method but with slight modification [22]. UVA irradiation was carried out using a UVA cross linker (CL-1000 L; Analytik Jena, CA, USA) with the wavelength set at 345 nm. Hs68 cells were cultured in 96-well plates (5 × 10^3^ cells/well) containing 10% FBS and 1% PSN for 24 h, then washed with 1× PBS twice to eliminate any residue. Next, Hs68 cells were covered with a thin layer of 1× PBS without a lid and then irradiated at doses of 5–20 J/cm^2^. The cell medium was subsequently replaced with serum-free DMEM and then incubated for 30 min, after which an MTT assay was performed to measure the Hs68 cell viability.

### 2.5. Cell Viability

Cell viability was determined via an MTT assay following a previously described procedure with slight modification [17,19]. Hs68 cells were seeded in 96-well plates (5 × 10^3^ cells/well) and allowed to adhere for 24 h. The cells were incubated with 200 μL of DMEM containing PNPs concentrations of 62.5, 125, 250, and 500 μg/mL. A group without PNPs was used as a control group. After incubation for 24 h, 3-[4,5-dimethyl-thiazol-2-yl]-2,5-diphenyltetrazolium bromide was dissolved in 1× PBS to prepare an MTT stock solution (5 mg/mL), which was then diluted with the DMEM. Next, 100 μg/mL MTT solution was added to the 96-well plates and incubated under 5% CO_2_ at 37 °C for 2 h. Then, 200 μg/mL dimethyl sulfoxide (DMSO) was added to each well, and the absorbance was measured at 570 nm using an ELISA reader. Cell viability was calculated using the following equation:

Cell viability (%) = [(OD_570_(sample)/OD_570_(control))] × 100% (2)

where OD_570_(sample) represents the absorbance of the sample, and OD_570_(control) represents the absorbance of the control.

### 2.6. Determining the Protective Ability of PNPs against UVA-Induced Cell Aging

Hs68 cells were seeded in 96-well plates (5 × 10^3^ cells/well) for 24 h. After the cells had adhered to the plate, 200 µL of DMEM containing PNPs (62.5, 125, 250, and 500 μg/mL) was added to the wells. The group without PNPs acted as a control group. Cells were washed with 1× PBS twice to remove any residual medium and then covered with a thin layer of 1× PBS for irradiation under UVA at a dose of 5 J/cm^2^. After UVA irradiation, the cells were washed twice with 1× PBS, after which 100 μL of MTT solution was added (500 μg/mL per well) before the samples were left to incubate for 2 h. Finally, 200 μL of DMSO was added and the absorbance was measured at 570 nm to evaluate the cell viability [22].

### 2.7. Morphological Analysis

Hs68 cells were seeded in 96-well plates for cell adherence. PNPs were then added at concentrations of 62.5–500 μg/mL and incubated for 24 h. Next, the cells were washed twice with 1× PBS and their morphological changes were observed using a fluorescence microscope (Olympus IX51, Tokyo, Japan) [23].

### 2.8. ROS Measurement

Hs68 cells were seeded in 96-well plates (8 × 10^5^ cells/well) and allowed to adhere for 24 h. The cells were incubated with PNPs (250 μg/mL) for 24 h and then exposed to UVA (5 J/cm^2^) for 30 min. After pretreatment, the Hs68 cells were incubated in the dark in serum-free DMEM containing H2DCF-DA (10 μM) at 37 °C for 30 min. After removal of the probe and washing of the samples twice with PBS, the final results were examined using a fluorescence microscope. The mean fluorescence density values were analyzed using Image J software [19].

### 2.9. β-Galactosidase Staining

Hs68 cells were seeded in 12-well plates (5 × 10^5^ cells/well) under 5% CO_2_ at 37 °C for 24 h, followed by UVA irradiation (5 J/cm^2^). An SA-β-gal kit (Cell Signaling Technology, Danvers, MA, USA) was employed to stain the Hs68 cells according to the manufacturer’s instructions. Cells were observed using a fluorescence microscope (Olympus IX51) and then quantified using Image J software [24,25].

### 2.10. Western Blot

Hs68 cells were pretreated with PNPs (250 μg/mL) and retinoic acid (RA, 10 μM), followed by UVA irradiation at a dose of 5 J/cm^2^. Protein expression was detected via Western blot analysis. Cells were collected and washed with 1× PBS and then lysed in 1× RIPA buffer to obtain whole-cell lysates. The supernatant was collected for protein quantification using a Bradford protein assay, and BSA was used for standard curve determination. Cell lysates were separated using sodium dodecyl sulfated polyacrylamide gel electrophoresis (SDS-PAGE), and the protein was transferred to polyvinylidene difluoride membranes. Afterward, the membrane was blocked with 5% nonfat milk in TBST buffer for 1 h and then treated with appropriate primary antibodies overnight at 4 °C, namely MMP-1, 3, 9 (1:1000), and GADPH (1:10,000). Finally, the membrane was washed three times with TBST (5 min each wash) to remove any primary antibodies, and it was then incubated with the corresponding conjugated secondary antibodies for 1 h at room temperature. Immunoreactive proteins were visualized via ECL chemiluminescent detection (Amersham Biosciences, Buckinghamshire, UK) and analyzed using GeneSnap software with the GeneTools image analyzer (Syngene, Cambridge, UK) [23,26,27]. All western blot assays were performed three times independently. The band intensities of each blot were quantified by Image J software. After normalized with the intensity of housekeeping protein (GAPDH), all data were versus with control group data, and then subjected to statistical analysis.

### 2.11. Nuclear Magnetic Resonance

To obtain the nuclear magnetic resonance (NMR) spectra, 20 mg of dried PNPs powder was dissolved in 1 mL of D_2_O. An Agilent 400-MHz NMR spectrometer was used to record 1H, 13C, HSQC, and HMBC NMR spectra at room temperature with standard pulse sequences. A JEOL 400-MHz NMR spectrometer was employed to record the NMR spectra of HSQC and HMBC at room temperature with standard pulse sequences [28]. 

### 2.12. Statistical Analysis

All results are expressed as the mean ± standard deviation (SD). Statistical data processing was implemented through dispersion analysis with SPSS 20 software. Statistical analysis was performed using one-way ANOVA and Duncan’s multiple range tests. *p* values of <0.05 were considered to indicate statistical significance [19].

## 3. Results

### 3.1. Inhibition of Elastase Activity by PNPs

**Figure 1:**
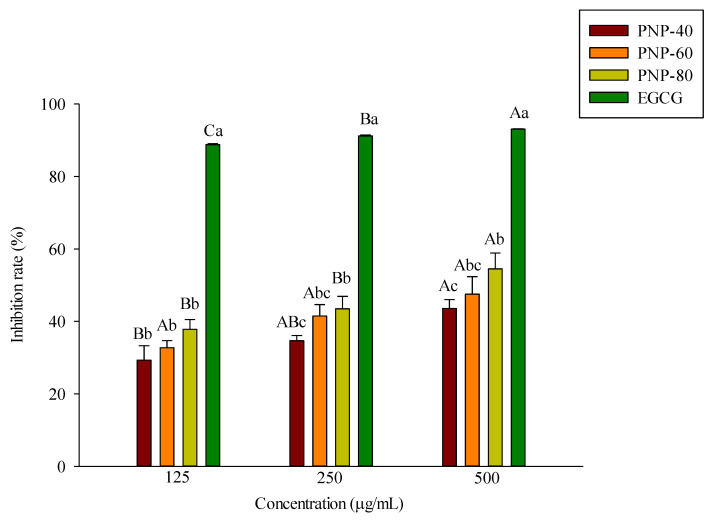
Anti-elastase activity of PNPs (125, 250, and 500 μg/mL) and EGCG (125, 250 and 500 μg/mL). Experiments were conducted in triplicate (*n* = 3). Data are expressed as the mean ± SD. a–c: Matching lowercase letters between samples at the same concentration indicate significant differences between the samples at that concentration (*p* < 0.05). A–C: Matching uppercase letters for the same sample at different concentrations indicate significant differences for that sample between different concentrations (*p* < 0.05).

Oxidative stress, UV rays, and cytokines degrade elastase, collagenase, and other components of the dermal ECM. Elastase can degrade elastin in the ECM, which can then cause skin wrinkle formation [29]. We investigated the ability of PNPs to repress elastase activity. An in vitro anti-elastase activity assay was adopted, with EGCG acting as a positive control. Figure 1 shows the elastase inhibitory activity of PNPs and EGCG. All samples exhibited an inhibitory effect in a dose-dependent manner from 125 to 500 μg/mL. The results showed that at a concentration of 500 μg/mL, the inhibitory rates of PNP-40, PNP-60, and PNP-80 were 43%, 47%, and 54%, respectively, and the inhibitory rate of EGCG was 93%. This suggested that PNPs can inhibit elastase activity. 

### 3.2. Effect of PNPs on Hs68 Cell Viability

**Figure 2:**
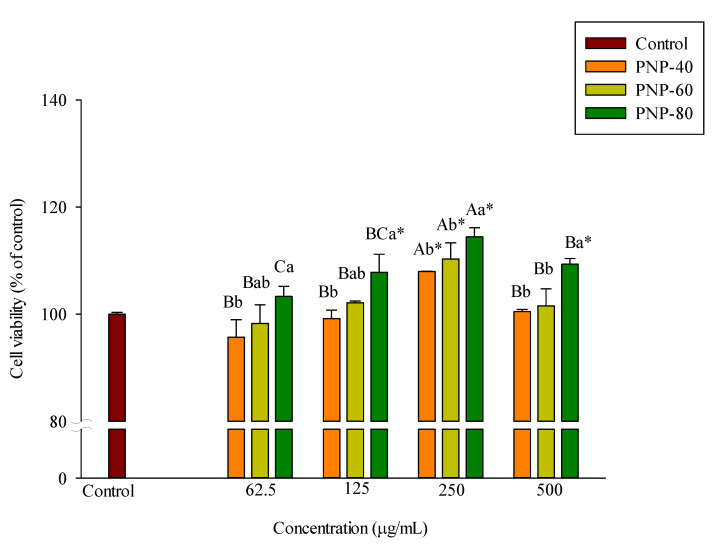
Cell viability analyzed via MTT assay. Cell viability was assessed using 3-(4,5-dimethylthiazolyl-2) 2,5 diphenyltetrazolium bromide (MTT) assays. Hs68 cells were treated with PNPs for 24 h. Experiments were conducted in triplicate (*n* = 3). Data are expressed as the mean ± SD. * Significant differences compared to the control group (*p* < 0.05). a,b: Matching lowercase letters between samples at the same concentration indicate significant differences between the samples at that concentration (*p* < 0.05). A–C: Matching uppercase letters for the same sample at different concentrations indicate significant differences for that sample between different concentrations (*p* < 0.05).

Cells were incubated with various PNPs concentrations for 24 h, after which the cellular density and changes in condition were observed. Finally, cell viability was assessed using an MTT assay. The results in Figure 2 show that at all concentrations, PNP-40, PNP-60, and PNP-80 had no adverse effects on the viability of the Hs68 cells. 

### 3.3. UVA Exposure Dose-Dependently Reduces Fibroblast Viability 

**Figure 3:**
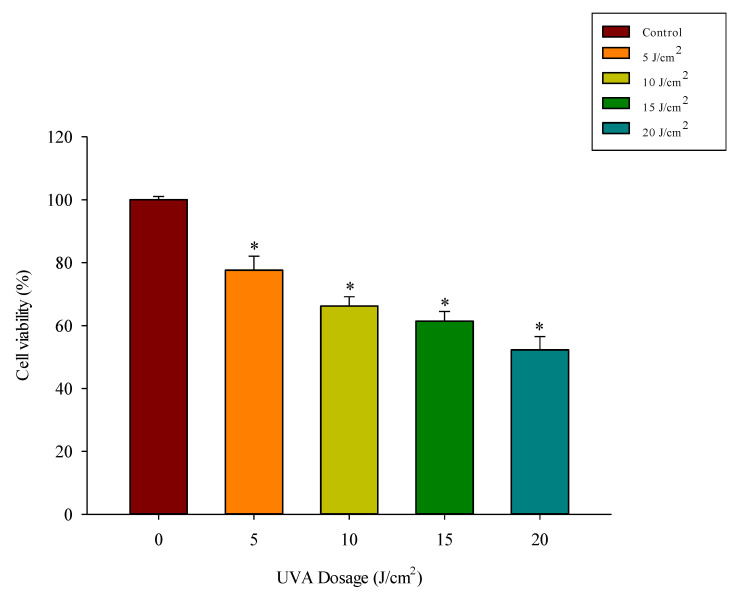
Effect of UVA irradiation on cell viability. Cells were irradiated with the indicated single doses of UVA radiation (5–20 J/cm^2^). Viability was determined via MTT assay at 24 h. Experiments were conducted in triplicate (*n* = 3). Data are expressed as the mean ± SD. * Significant differences compared to the control group (*p* < 0.05).

Hs68 cells were irradiated with UVA at a dose of 5–20 J/cm^2^, after which cell viability was measured using an MTT assay. The results in Figure 3 show that increasing the UVA dose decreased the cell viability to 77%, 66%, 61%, and 52% at doses of 5, 10, 15, and 20 J/cm^2^, respectively (*p* < 0.05). Previous research reported a significant increase in MMP-1 expression when the UVA dose was increased to 5 J/cm^2^ from 3 J/cm^2^ [30]. Also in that study, after irradiation with more than 5 J/cm^2^, the cell viability decreased. Therefore, we used 5 J/cm^2^ as the irradiation dose in the present study. 

### 3.4. PNPs Protect Fibroblasts against UVA-Induced Cell Death

**Figure 4:**
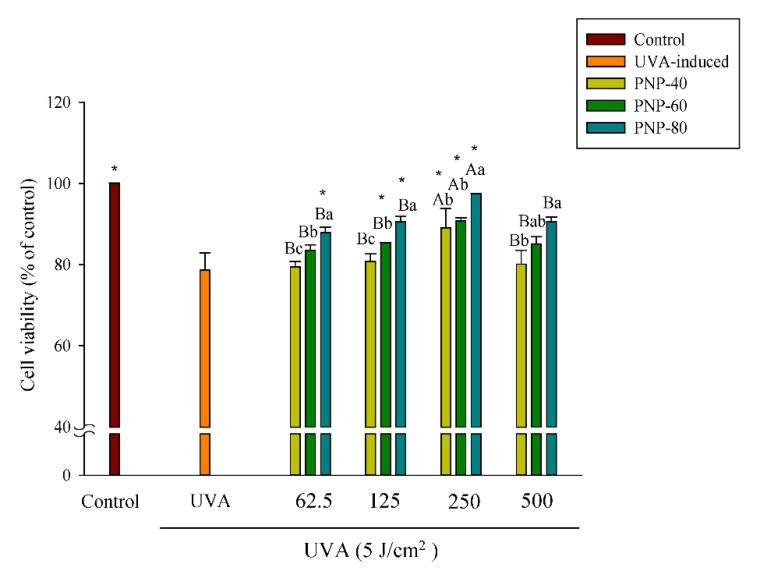
Cell viability of Hs68 cells pretreated with PNPs (62.5, 125, 250, and 500 μg/mL) for 24 h and then subjected to UVA irradiation at 5 J/cm^2^, as determined via MTT assay. Experiments were conducted in triplicate (*n* = 3). Data are expressed as the mean ± SD. * Significant differences compared to the UVA-induced group (*p* < 0.05). a–c: Matching lowercase letters between samples at the same concentration indicate significant differences between the samples at that concentration (*p* < 0.05). A,B: Matching uppercase letters for the same sample at different concentrations indicate significant differences for that sample between different concentrations (*p* < 0.05).

The controls and UVA-induced group were contained only in DMEM, with or without UVA irradiation. As shown in Figure 4, the cell viability of the UVA-irradiated group was 78% when pretreated with PNPs (62.5–500 µg/mL) for 24 h and then irradiated with UVA at a dose of 5 J/cm^2^. The results in the figure also show that the cell viability of PNP-80 at concentrations from 62.5 to 250 µg/mL was significantly higher than that in the UVA-induced group (87%, 90%, and 97%, respectively). For the samples pretreated with PNP-40 and PNP-60 and then irradiated with UVA at a dose of 5 J/cm^2^, the cell viability at 250 µg/mL showed a significantly larger protective effect compared to the UVA-induced group; however, the cell viability of PNPs at 500 µg/mL was slightly lower than that observed at 250 µg/mL. In summary, UVA irradiation decreased cell viability and induced morphological changes. PNPs exhibited a protective effect against UVA-induced oxidative damage in the Hs68 cells; at a concentration of 250 µg/mL, pretreatment with PNPs resulted in significantly higher cell viability compared to the UVA-induced group, indicating that they might promote cell proliferation. Therefore, 250 µg/mL was adopted as the dose in our subsequent experiments.

### 3.5. Effect of PNPs on UVA-Induced ROS Production in Hs68 Cells

**Figure 5:**
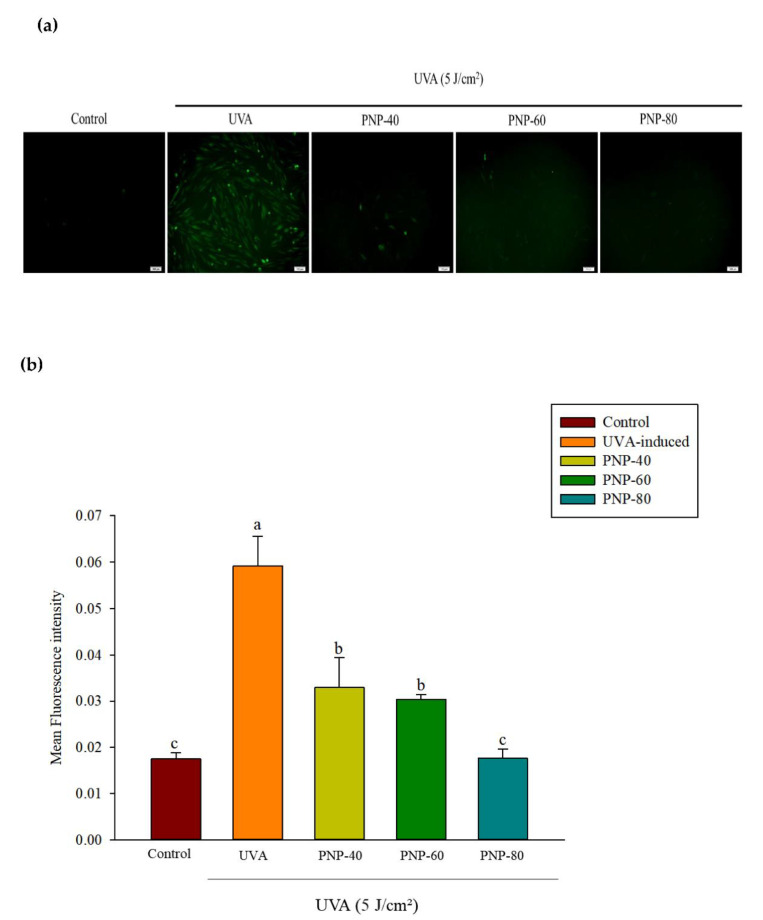
(**a**) Radical scavenging activity of PNPs (250 µg/mL) on Hs68 cells against UVA-induced ROS generation (scale bar = 100 µm, magnification: 10 × 10). (**b**) Quantitative analysis (performed using Image J software) of the radical scavenging effect of PNPs on Hs68 cells against UVA-induced ROS generation. Experiments were independently conducted in triplicate (*n* = 3). Data are expressed as the mean ± SD. a–c: Matching lowercase symbols indicate significant differences between the different groups (*p* < 0.05).

The results indicated that PNPs showed no cytotoxicity toward Hs68 cells at 250 μg/mL and that they can protect Hs68 cells from UVA-induced cell damage. Cells were first pretreated with PNPs (250 µg/mL) and then exposed to continuous UVA irradiation at 5 J/cm^2^. ROS levels were then determined via H2DCFH-DA staining. In Figure 5a, the green fluorescence indicates ROS production and intensity, which was quantified using Image J software (Figure 5b). As shown in Figure 5, irradiation with UVA at 5 J/cm^2^ significantly increased the ROS fluorescence intensity to 0.059; however, pretreatment with PNP-40, PNP-60, and PNP-80 resulted in a significant decrease to 3.3%, 3.0%, and 1.7%, respectively. Pretreatment with PNP-60 and PNP-80 showed no significant difference compared with the control group. 

### 3.6. Effect of PNPs on UVA-Induced Senescence in Hs68 Cells 

**Figure 6:**
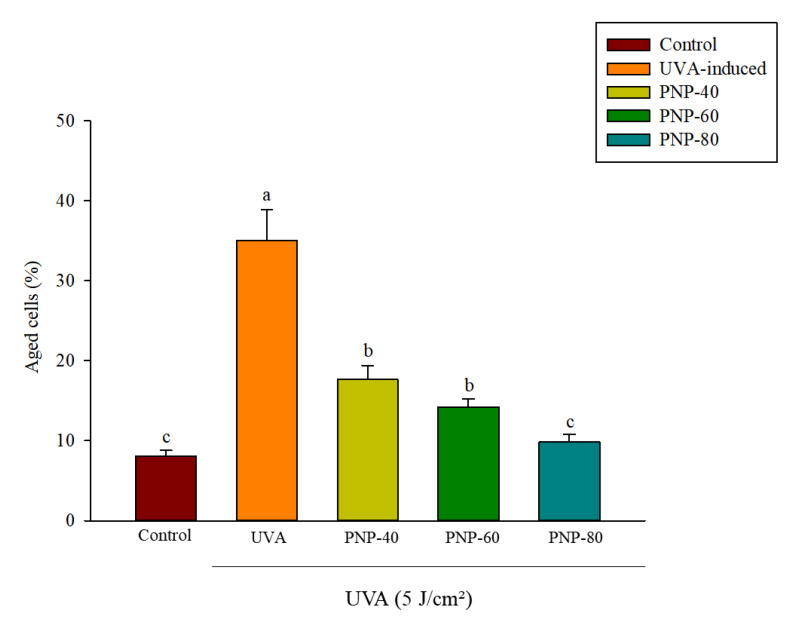
Protective effect of PNPs against cellular senescence in Hs68 cells irradiated with UVA. Experiments were conducted in triplicate (*n* = 3). Data are expressed as the mean ± SD. a–c: Matching lowercase letters indicate significant difference between the different groups (*p* < 0.05). Experiments were conducted in triplicate (*n* = 3). Data are expressed as the mean ± SD.

Hs68 cell senescence might delay ECM secretion and formation [31]. Therefore, β-galactosidase staining was adopted to analyze whether PNPs can inhibit Hs68 cell aging. The control and UVA groups were treated only with DMEM. The concentration of the PNPs group was 250 µg/mL. Only the control group was not irradiated with UVA; the remaining groups were irradiated at a dose of 5 J/cm^2^. Figure 6 shows the quantification results obtained using Image J software. The percentage of aged cells increased significantly to 35% after UVA irradiation; however, for those treated with PNP-40, PNP-60, and PNP-80, this was reduced to 17%, 14%, and 9%, respectively. Previous research showed that UVB irradiation can induce aging in fibroblast cells, and that GL-PS treatment (40 μg/mL) can inhibit UVA-induced aging of fibroblast cells. 

### 3.7. PNPs Downgrade UVA-Induced MMP-1, -3, -9 Expression in Hs68 Cells 

**Figure 7:**
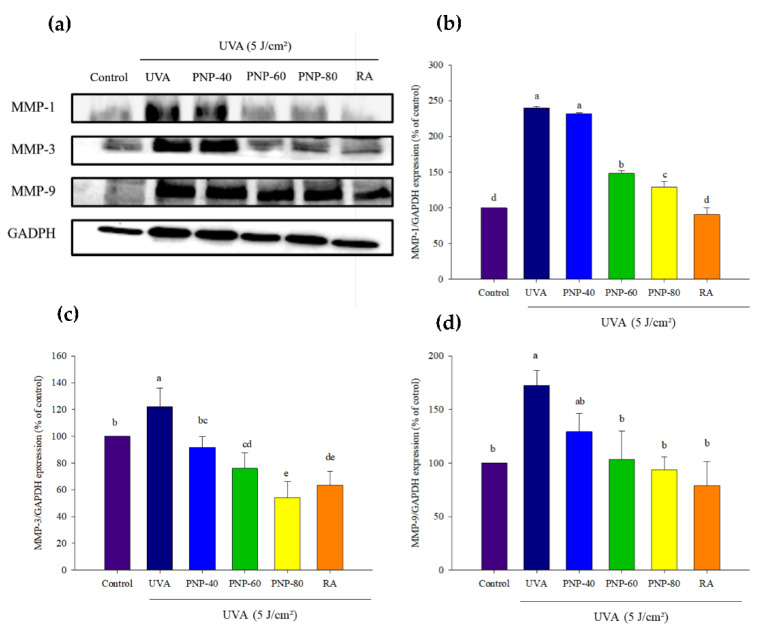
Protective effect of PNPs against UVA irradiation in Hs68 cells. Protein expression of MMP-1, -3, and -9 was analyzed via Western blot. (**a**) Hs68 cells were pretreated with PNPs (250 µg/mL) and RA (10 µM) for 24 h, followed by UVA-irradiation. Western blotting was performed to examine MMP-1, -3 and -9 expression, with GAPDH used as an internal control. (**b**–**d**) MMP-1, -3, and -9 expression after quantification using Image J software. a–e: Matching lowercase letters indicate significant differences between the different groups (*p* < 0.05). Experiments were conducted in triplicate (*n* = 3), and the blot shown was the representative result. Data are expressed as the mean ± SD.

We evaluated whether PNPs could diminish MMP-1, -3, -9 expression in Hs68 cells. For the cells grown in DMEM, the PNPs groups were pretreated with PNPs at 250 µg/mL. An RA group treated with retinoic acid (RA) at 10 µM was incubated for 24 h. These groups were irradiated with UVA at a dose of 5 J/cm^2^. Figure 7a shows the MMPs expression results, which were analyzed via Western blotting and quantified using Image J software. The MMP-1 level was significantly increased to 223% after UVA irradiation, but in treatment with PNP-40, PNP-60, PNP-80, and RA, the MMP-1 level was 218%, 126%, 116%, and 55%, respectively. The MMP-3 level increased to 115% after UVA irradiation, but treatment with PNP-40, PNP-60, PNP-80, and RA reduced this to 80%, 62%, 54%, and 63%, respectively. The MMP-9 level increased significantly to 185% after UVA irradiation, but treatment with PNP-40, PNP-60, PNP-80, and RA reduced this to 162%, 146%, 126%, and 114%, respectively. 

### 3.8. NMR 

**Figure 8:**
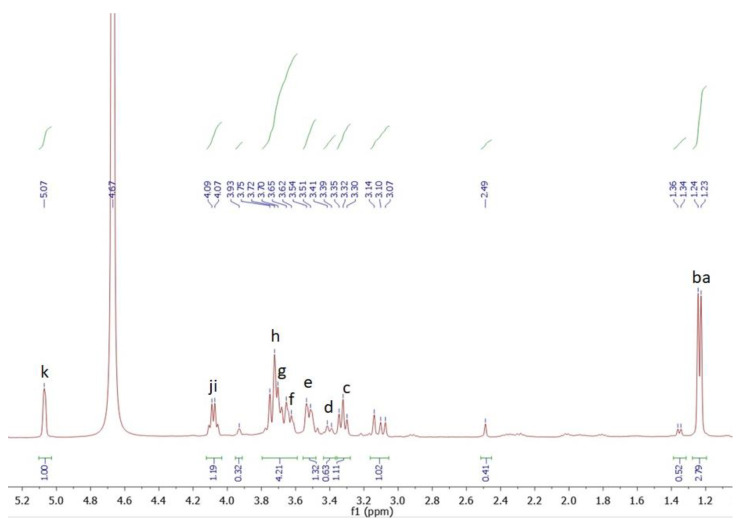
1H NMR spectroscopy of PNP-80. a–k: Matching lowercase letters represent hydrogen signals at different positions on the PNP-80 structure.

**Figure 9:**
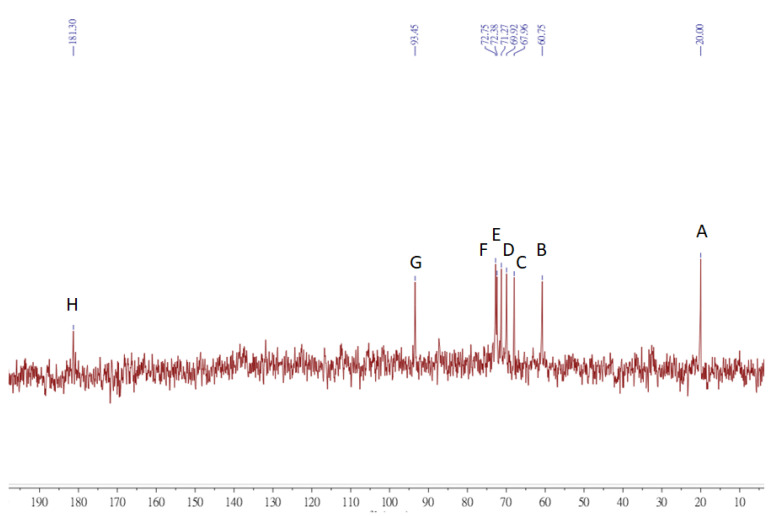
13C NMR spectroscopy of PNP-80. A–H: Matching capital letters represent carbon signals at different positions on the PNP-80 structure.

In this study, we compared the ability of inhibiting photoaging from PNP-40, PNP-60, and PNP-80. We found that PNP-80 was better than others for inhibiting photoaging. Therefore, we analyzed the structural characteristics of PNP-80 via 1-D NMR. Figure 8 and Figure 9 show the 1H and 13C NMR spectra of the PNPs. The multiple signals at δ 3.07–3.75 ppm are the characteristic signals of polysaccharides. There were the two main anomeric proton signals at δ 4.07–5.3 ppm, indicating that the PNPs were mainly composed of two types of pyran glucose of α- and β-configurations. The anomeric carbon signals (20, 60.75, 68, 70, 71, 72, 93.45, and 181.3 ppm) appear in identical 13C NMR spectra. Additionally, we examined the 2D NMR spectra of HSQC and HMBC, as shown in Figures S2 and S3. Also PNP-40, PNP-60 1-D NMR spectra as shown in Figures S4–S7. The proton and carbon signals of the PNPs correspond to the pyran glucose of α- and β-configurations (Table S1) [32]. The findings suggest that PNP-80 is a mixture of alpha-glucose, beta-glucose, and mannose. Previous studies have reported a similar pattern of the polysaccharide fraction PNP-80 [17,33].

## 4. Discussion

Elastin and collagen are the major components of the ECM in the dermis, and these are responsible for maintaining skin structure and elasticity while also playing a critical role in wound healing [34]. ROS elevate elastase activity, which then degrades elastin and leads to decreased skin thickness, elasticity, and poor water retention in the skin, resulting in wrinkle formation [11,35]. Thus, elastase inhibitors are crucial reagents that can delay and reduce skin aging and wrinkle formation. Previous research on *Porphyridium cruentum* polysaccharides has shown that it has a 46% inhibitory rate against elastase activity [21]. Other studies have reported that *Phellinus vaninii* extracted with hot water and methanol respectively showed elastase inhibitory rates of 28.9% to 64.7% and 37.2% to 71.2% at concentrations of 125 to 2000 μg/mL [36]; while *Coriolus versicolor* extracted using the same methods showed elastase inhibitory rates of 13.8% to 16.9% and 11.4% to 32.9% at concentrations of 1000 to 3000 μg/mL [37]. In this study, PNP-40, PNP-60, and PNP-80 exhibited elastase inhibitory rates of 29% to 43%, 35% to 47%, and 37% to 54%, respectively, which is higher than that achieved with polysaccharides extracted from *C. versicolor* and *P. vaninii*. Another study reported that PNPs exhibited a high level of anticollagenase activity, with PNP-80 showing a more favorable inhibitory rate than EGCG [19]. In the present study, we also found that PNP-80 achieved the best elastase inhibition activity. In summary, the collective results of these studies indicate that PNPs are effective elastase and collagenase inhibitors that could reduce elastin degradation and delay skin wrinkle formation.

UV radiation produces excessive ROS through increased NADPH oxidase enzyme activity, which generates superoxide anions (O^2−^) and other ROS. Second, UV irradiation reacts with porphyrin and then produces singlet oxygen (_1_O^2^) followed by conversion to other ROS [11,12]. Excessive ROS content activates signaling pathways such as MMPs expression, which can exacerbate degradation of the ECM and cause skin wrinkle formation [38,39]. UV irradiation is the main external factor that causes the skin to age and wrinkle [40,41,42]. Excessive ROS formation due to UV irradiation decomposes cell morphology, influences ECM secretion, and causes loss of other functions before finally inducing cell apoptosis [9,10]. Previous studies have indicated that exposure to UVA (320~400 nm) can injure the skin matrix and cause wrinkles, leading to skin aging [11]. The results of the present study indicated that PNPs precipitated by fractional ethanol can ameliorate UVA-induced oxidative stress, with PNP-80 showing the largest effect against ROS production, but with no significant difference from the control group. Ultraviolet–visible absorption spectra of PNPs show that PNP-40, PNP-60, and PNP-80 displayed a maximum absorption peak of around 200 nm (Figure S1), which was similar to the characteristics of Ultraviolet–visible absorption spectra of natural polysaccharides as previously reported [43,44,45]. Therefore, UVA absorption (320~340 nm) may not be the main mechanism underlying the protective effect of PNPs on UVA-induced photoaging in Hs68 cells. These results support the findings of previous research, proving that PNPs can ameliorate H_2_O_2_-induced oxidative stress; furthermore, no cytotoxicity toward L929 cells was observed [17,19]. Previous investigations have indicated that PNPs can elevate superoxide dismutase, catalase, and glutathione peroxidase antioxidant enzyme activity [46,47]. This also suggests that the mechanism of antioxidant activity between polysaccharides and polyphenol are similar; specifically, they mainly transfer the H atom through hydroxyl and carbonyl groups. In addition, the presence of a polar R group in proteins and amino acids might have potential for scavenging ROS [48]. Another previous study reported that UVA exposure at a dose of 30 J/cm^2^ significantly increased ROS production in HaCaT cells, and pretreatment with *Astragalus membranaceus* polysaccharides (200 μg/mL) significantly inhibited ROS production [49].

After UV irradiation, the skin texture becomes rough and dry with deep wrinkling and loss of function to retain water in the skin. Excessive ROS induced from UV irradiation triggers different cell signaling pathways to activate MAPKs and NF-κB, which trigger MMPs expression. Matrix metalloproteinases appear to have a function in ECM degradation, with MMP-1, -3, and -9 breaking down most of the ECM in the dermis, which results in a loss of skin elasticity [50,51]. MMPs are endopeptidases, which need zinc ions as cofactors, and they play a critical role in photoaging [48,52]. The results of this study demonstrate that MMP-1, -3, and -9 expression increased after UVA irradiation but decreased in the PNPs pretreatment and RA groups. Previous research pointed out that PNPs could chelate metal ion Fe^2+^, which might chelate the metal ion zinc [17]. Another previous study indicated that *G. lucidum* polysaccharides could reduce UVB irradiation-induced ROS content to downregulate MMP-1 levels and promote collagen expression [25].

## 5. Conclusions

UV irradiation is the main factor contributing to skin photoaging and wrinkle formation because it damages substances in human dermal fibroblasts and causes strand breaks in mitochondrial DNA, which will generate excessive ROS [39,41,50,51,53,54]. The results of the present study indicate that PNPs are excellent elastase inhibitors and could reduce elastin degradation and delay skin wrinkle formation. The experimental results showed that PNPs can efficiently eliminate UVA-induced excessive ROS as well as MMP-1, -3, and -9 content in Hs68 cells. Furthermore, we found that PNP-80 is a mixture of alpha-glucose, beta-glucose, and mannose with NMR, and PNP-80 has the best ability to protect Hs68 cells against UVA-induced photoaging. In summary, the findings of both the present study and previous studies suggest that PNPs might be effective antiphotoaging reagents and could be developed as functional cosmetic materials. However, further research is necessary to determine how PNPs regulate photoaging and inflammatory signaling pathways.

## Data Availability

Data is contained within the article and Supplementary Material.

## Supplementary Materials

The following supporting information can be downloaded at: https://www.mdpi.com/article/10.3390/antiox11040739/s1, Figure S1. Ultraviolet–visible absorption spectra of PNP-40, PNP-60, and PNP-80; Table S1. Assignment of 1H and 13C NMR spectra for PNP-80; Figure S2. The HSQC spectra of PNP-80; Figure S3. The HMBC spectra of PNP-80; Figure S4. 1H NMR spectroscopy of PNP-40; Figure S5. 13C NMR spectroscopy of PNP-40; Figure S6. 1H NMR spectroscopy of PNP-60; Figure S6. 1H NMR spectroscopy of PNP-60; Figure S7: 13C NMR spectroscopy of PNP-60.
