# Supplementary material for: Pholiota nameko Polysaccharides Protect against Ultraviolet A-Induced Photoaging by Regulating Matrix Metalloproteinases in Human Dermal Fibroblasts
Source: Antioxidants (Basel). 2022 Apr 8;11(4):739. doi: 10.3390/antiox11040739 (PMC9029720; doi:10.3390/antiox11040739)
Supplement: Supplementary file 1 [file antioxidants-11-00739-s001.zip › antioxidants-1612482-supplementary.pdf]

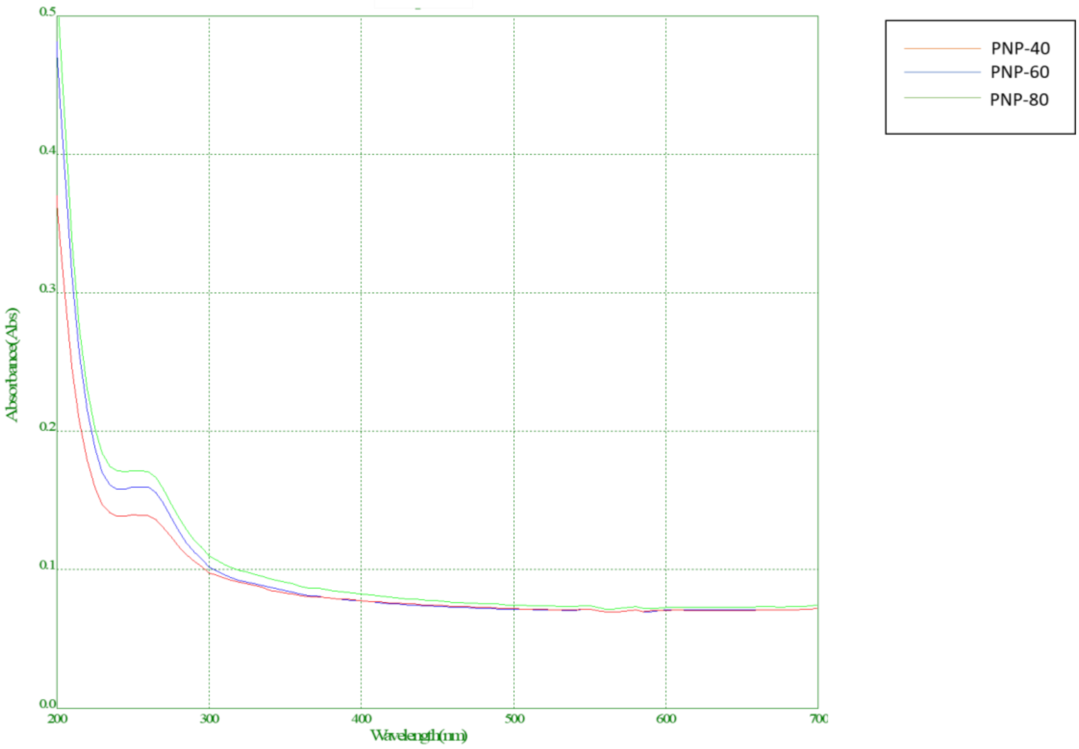

Figure S1. Ultraviolet–visible absorption spectra of PNP-40，PNP-60, and PNP-80.

Table S1. Assignment of 1H and 13C NMR spectra for PNP-80.

Peak of C spectrum	Chemical shift of C spectrum(ppm)	Peak of H spectrum	Chemical shift of H spectrum(ppm)
A	20	a	1.23
		b	1.24
B	60.75	f	3.62
		h	3.73
C	68	i	4.07
		j	4.09
D	70	c	3.3
E	71	d	3.4
		e	3.5
F	72	g	3.7
G	93.45	k	5.08
H	181.3		

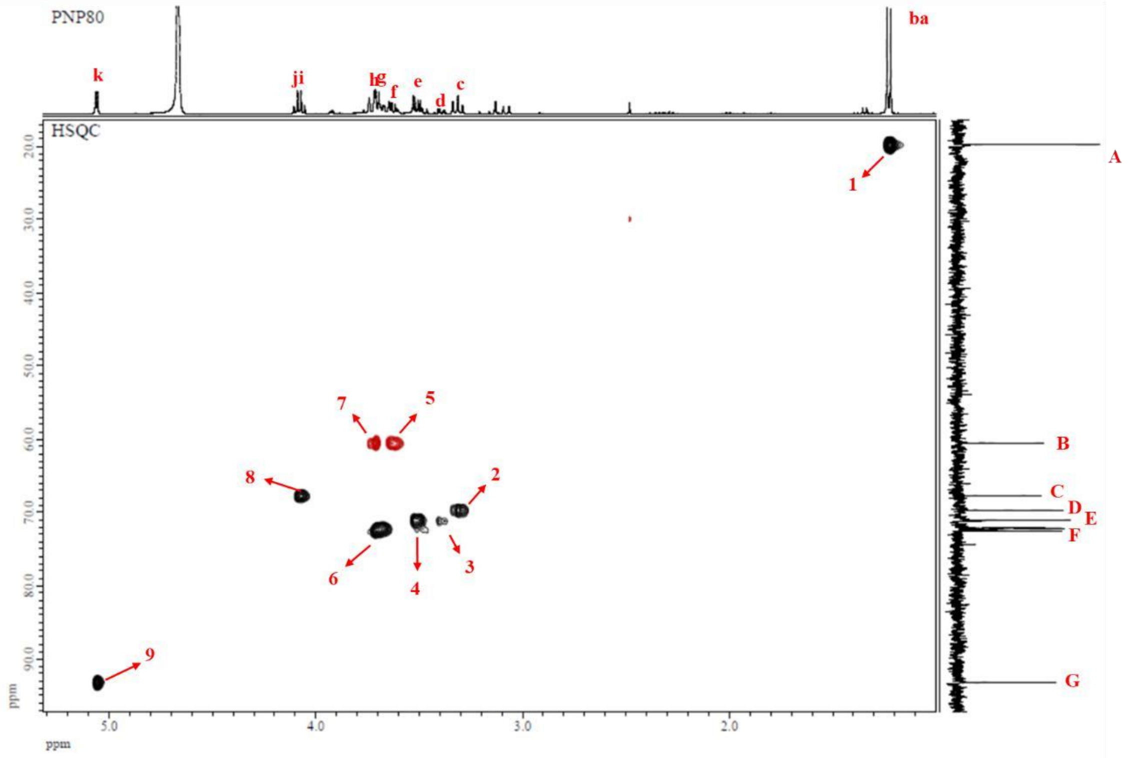

Figure S2. The HSQC spectra of PNP-80.

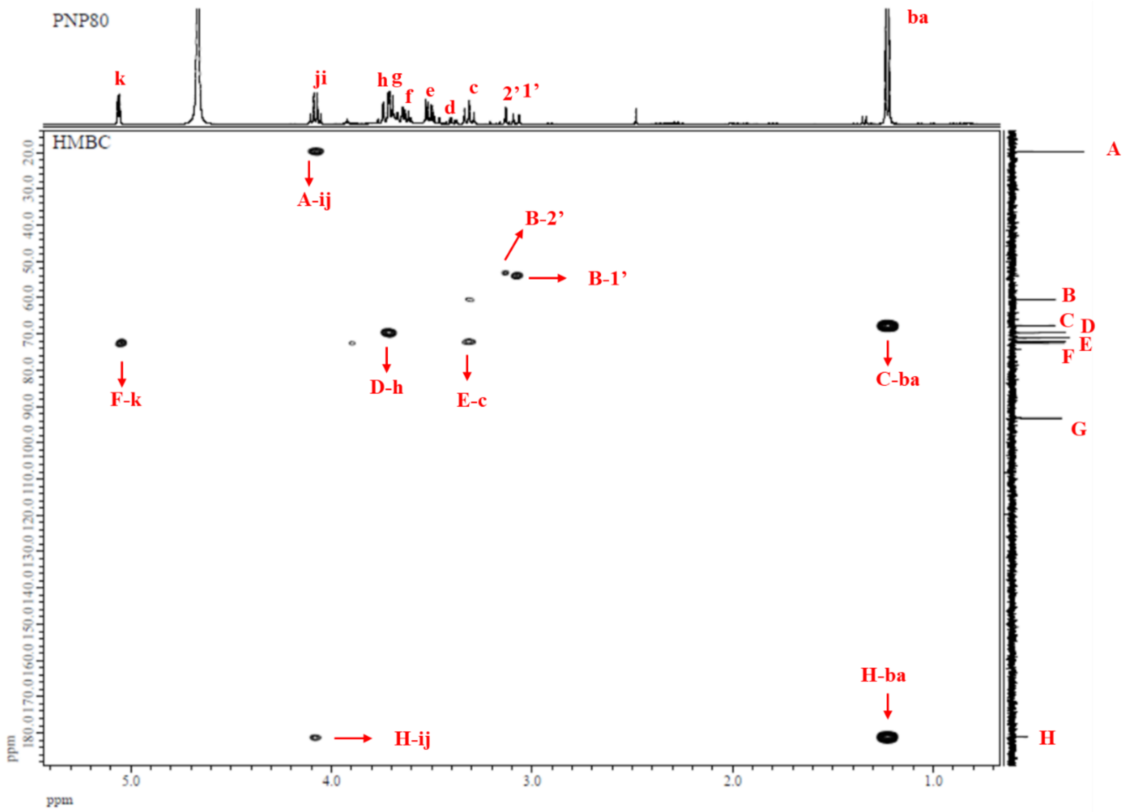

Figure S3. The HMBC spectra of PNP-80.

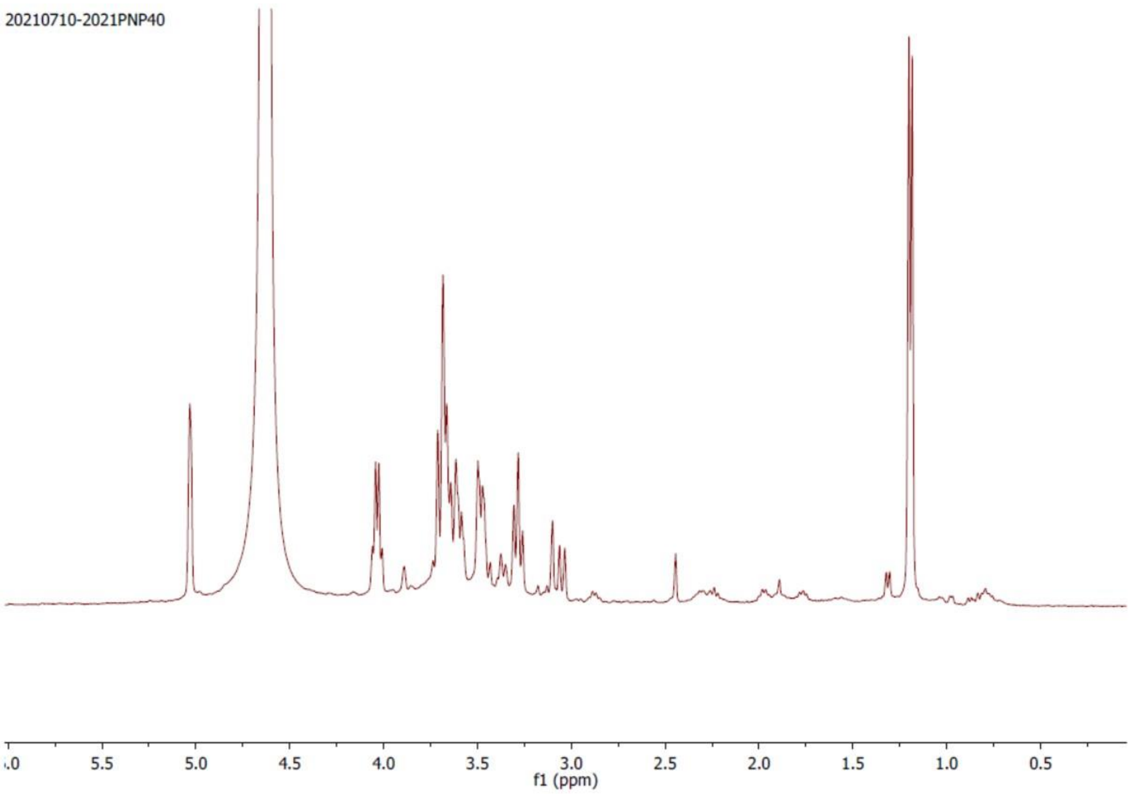

Figure S4. 1H NMR spectroscopy of PNP-40.

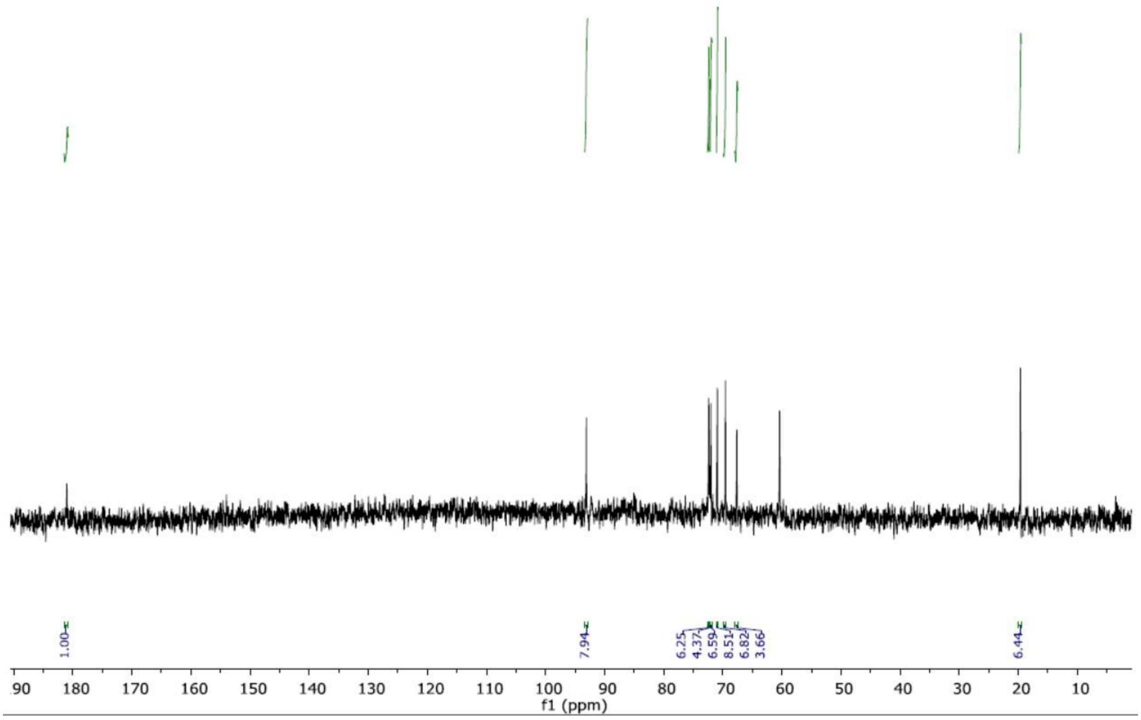

Figure S5. 13C NMR spectroscopy of PNP-40.

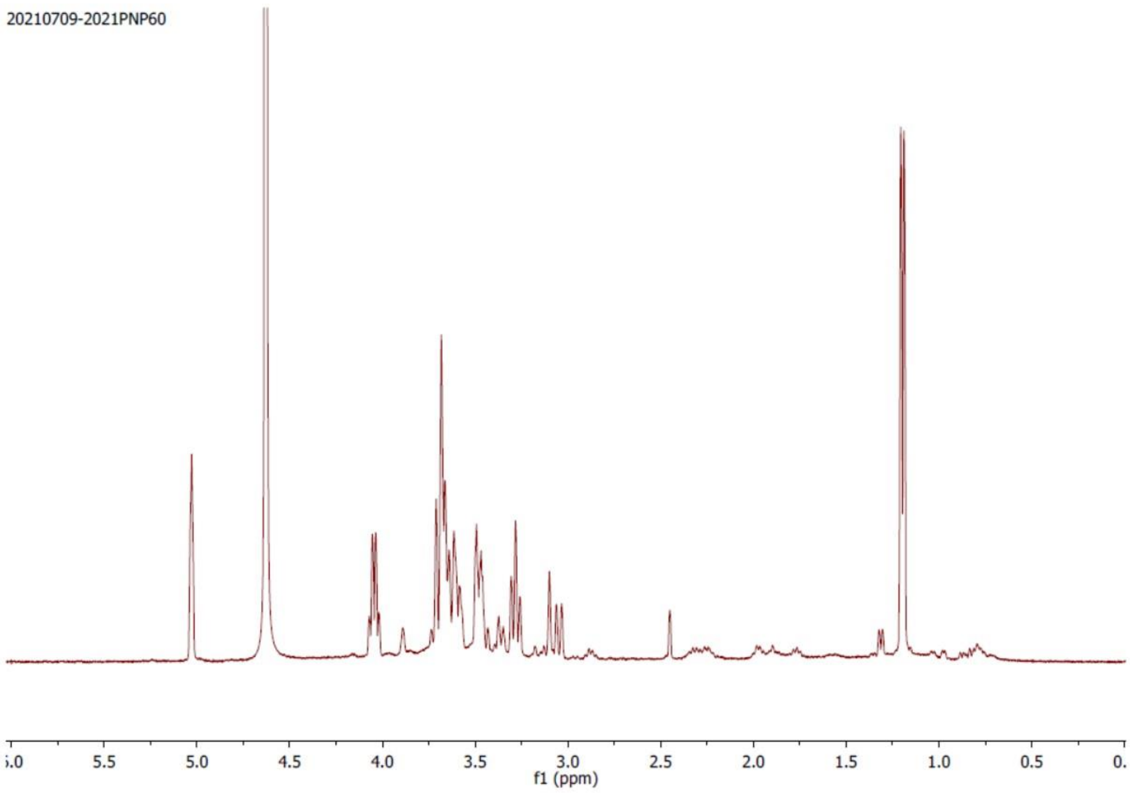

Figure S6. 1H NMR spectroscopy of PNP-60.

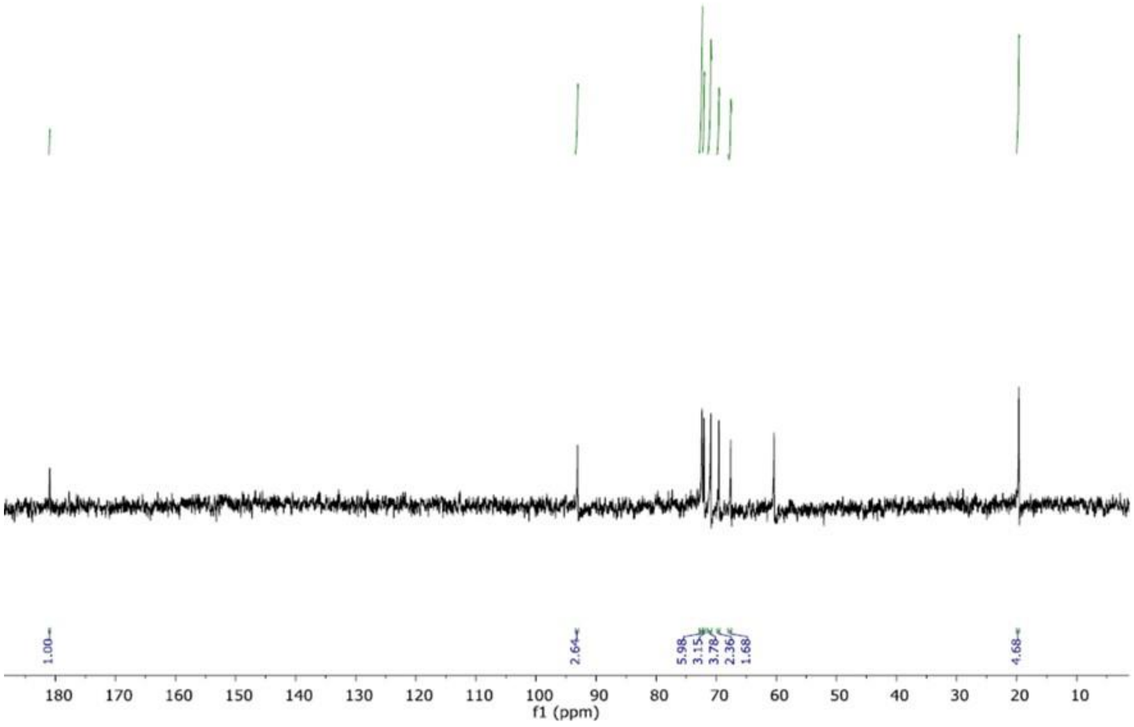

Figure S7: 13C NMR spectroscopy of PNP-60
